# Beyond cultural stereotyping: views on end-of-life decision making among religious and secular persons in the USA, Germany, and Israel

**DOI:** 10.1186/s12910-017-0170-4

**Published:** 2017-02-17

**Authors:** Mark Schweda, Silke Schicktanz, Aviad Raz, Anita Silvers

**Affiliations:** 1 Department of Medical Ethics and History of Medicine, University Medical Center Göttingen, Humboldtallee 36, 37073 Göttingen, Germany; 2 Department of Sociology and Anthropology, Ben-Gurion University of the Negev, Be’er-Sheva, 84105 Israel; 3 Department of Philosophy, San Francisco State University, 1600 Holloway Avenue, San Francisco, CA 94132 USA

**Keywords:** Attitudes toward death, Advance directives, Assisted suicide, Euthanasia, Cultural diversity, Focus groups

## Abstract

**Background:**

End-of-life decision making constitutes a major challenge for bioethical deliberation and political governance in modern democracies: On the one hand, it touches upon fundamental convictions about life, death, and the human condition. On the other, it is deeply rooted in religious traditions and historical experiences and thus shows great socio-cultural diversity. The bioethical discussion of such cultural issues oscillates between liberal individualism and cultural stereotyping. Our paper confronts the bioethical expert discourse with public moral attitudes.

**Methods:**

The paper is based on a qualitative study comprising 12 focus group discussions with religious and secular persons in the USA, Germany, and Israel (*n* = 82). Considering the respective socio-political and legal frameworks, the thematic analysis focuses on moral attitudes towards end-of-life decision making and explores the complex interplay between individual preferences, culture, and religion.

**Results:**

Our findings draw attention to the variety and complexity of cultural and religious aspects of end-of-life decision making. Although there is local consensus that goes beyond radical individualism, positions are not neatly matched with national cultures or religious denominations. Instead, the relevance of the specific *situatedness* of religious beliefs and cultural communities becomes visible: Their status and role in individual situations, for example, as consensual or conflicting on the level of personal perspectives, family relationships, or broader social contexts, e.g., as a majority or minority culture within a political system.

**Conclusions:**

As the group discussions indicate, there are no clear-cut positions anchored in “nationality,” “culture,” or “religion.” Instead, attitudes are personally decided on as part of a negotiated context representing the political, social and existential situatedness of the individual. Therefore, more complex theoretical and practical approaches to cultural diversity have to be developed.

## Background

When Terri Schiavo died on March 31^st^ 2005, she had been at the centre of a protracted legal struggle and a heated public debate for 8 years. At the onset was a conflict between her husband and parents about removing her feeding tube after more than a decade in a persistent vegetative state. In the course of the controversy, however, the case engaged several levels of jurisdiction, the US-Congress, President George W. Bush, the US-Supreme Court, and even the Vatican. A great variety of political and ideological groups accompanied the whole process and voiced heterogeneous, at times completely incompatible views and agendas. Civil liberties movement representatives underlined the right to an autonomous decision and a dignified death [1]; Christian advocacy groups stressed the sanctity of human life and the absolute prohibition of killing [2]; Jewish commentators referred to Halakha to emphasize the moral difference between withholding and withdrawing treatment [3].

The fierce, at times seemingly irreconcilable culture wars sparked by the Schiavo case exemplify why end-of-life (EOL) decision making constitutes a major challenge for bioethical deliberation and political governance in modern liberal democracies. On the one hand, these decisions touch upon some of our most fundamental convictions regarding life and death, the meaning of being a human being and a person, and the principles of leading a good, decent and dignified life: How can we maintain the autonomy and dignity of dying patients in an environment dominated by professional paternalism and medical bureaucracy? Is it morally permissible to stop or withhold life sustaining treatment, assist terminally ill patients in committing suicide, or even conduct euthanasia? And to what extent can and should professional principles or political regulations intervene in these deeply personal and familial matters? On the other hand, however, social research indicates that public attitudes towards these issues are also deeply rooted in cultural, especially religious and spiritual traditions, as well as in specific historical experiences, and therefore often show great variety between different socio-cultural contexts [4–7].

The increasing sensitivity to cultural differences in bioethical debates on EOL decision making appears highly desirable [8]. Although there may be isolated attempts to exclude religious viewpoints, e.g., from relevant advisory committees, they frequently play a prominent role in the contemporary expert discourse [9]. At the same time, however, it is essential to develop adequate conceptions of culture and religion in order to avoid oversimplifying notions and cultural stereotyping. Indeed, many contributions to the debate still seem to express an insufficient, overly simplified picture by drawing on essentialist conceptions of culture and religion as monolithic, homogeneous entities with unchangeable defining features [10]. It is still common to identify “culture” with national characteristics or a religious denomination, or to equate religion with an official theological doctrine and expert discourse [11]. There are numerous popular publications claiming to present “the” Jewish, Christian, or Buddhist view on EOL [12, 13]. This simplified perspective may promote a kind of socio-cultural determinism. The assumption that being “American,” “German,” or “Israeli,” as well as being “Jewish,” “Christian,” or “secular,” automatically suggests particular positions towards EOL was already critically tested in comparative studies [14]. When used as a basis for guidelines for clinical practice or ethics consultation [15], or even as an input for algorithms of EOL decision making for incapacitated patients [16], such stereotypical understandings can yield highly problematic effects. The same holds true for political players who claim to speak on behalf of certain cultural or religious communities and to advocate their alleged positions [17].

In light of the longstanding controversial political and academic debate on liberalism and cultural pluralism in bioethics, there has been comparatively little empirical research on *actual* cultural specificities and differences in public attitudes towards medical EOL decision making [18]. Against the backdrop of the political and legal situation and the academic discourse, this study aims to explore similarities and differences in the moral perspectives of lay persons. For this purpose, we confront the bioethical and biopolitical expert discourse with concrete public views of and attitudes towards EOL decision making in different cultural settings. This consideration of “public understandings of ethics” allows a critical assessment of the plausibility and practicability of academic ethical arguments and political rationales [19]. The aim is to develop a more fine-grained understanding of cultural identity at the intersection of different religious and cultural commitments. Moreover, including the perspectives of affected persons who have already been in situations of EOL decision making can help to understand the perceptions and priorities of the patients themselves – a fundamental requirement for all ethical approaches that call for strengthening patient autonomy [19].

Our considerations are based on a qualitative socio-ethical study in the United States (California), Germany, and Israel. We begin with an overview of the bioethical expert discourses on EOL decision making and the pertinent practical, socio-political, and legal frameworks in the three countries, pointing out similarities as well as differences. Against this background, we analyse similarities and differences in public attitudes towards EOL decision making, with a focus on advance directives (AD), termination of life-sustaining treatment and euthanasia, as well as the role of the state. This research design allows us to examine the complex interplay between the diverse influences and interpretations of culture and religion. We will discuss our findings in light of the bioethical expert discourse on EOL and draw conclusions for professional decision making.

## Background: synopsis of the EOL-debate in the USA, Germany, and Israel

EOL decision making has been at the centre of bioethical discussions for several decades [20]. Especially in the USA, the call for more consideration of culture and religion in bioethics has received attention in the broader context of multiculturalism. Different cultural backgrounds and religious denominations have presented their stances [21–23], revealing a great diversity of positions [8, 24, 25]. Against this background, it has been suggested that in the spirit of culturally sensitive medical care, each group should have the right to be treated according to their respective worldviews and value systems [15]. However, once we step beyond our particular cultural communities and enter the public and political sphere, we seem to lack a consensual basis for formulating generally binding legal regulations [22]. A similar situation occurs on an international level: Even modern liberal democracies offering high standards of medical technology and care for EOL show at the same time important differences with regard to the public debate and the legal regulation of EOL decision making. In the following, we give a brief overview of the most important pertinent discussions and legal regulations in the USA (California), Germany, and Israel in order to provide a background and frame of reference for the empirical analysis of lay perspectives in all three countries (see [26]).

In the US, questions of EOL form part of the culture wars between liberal pro-choice groups and primarily Christian pro-life groups. The case of Karen Ann Quinlan in 1975 was at the onset of the recent political and public controversy and proved precedential for subsequent legislation and jurisdiction. Several federal states implemented legal regulations regarding the right to die and the conditions for the termination of life-prolonging treatment and physician-assisted suicide. The state of California played a particular role in this development. The *California Natural Death Act* (1977) was the first of its kind in the US. It allows directives instructing physicians not to use artificial methods to extend the natural dying process. A series of court rulings increased the range of physician obligations to obtain patient consent to treatment, expanded informed consent to include the risks of rejecting recommended procedures, and determined that life-sustaining treatment may not be stopped without evidence that the patient is in a persistent vegetative state and wants termination. Since 1991, the *Federal Patient Self-Determination Act* has required medical and care providers to inform patients in writing about their rights to participate in decisions concerning their medical care and about the provider’s policy on ADs, to include information in patients’ records about whether they have executed ADs, and to educate their staff and community about ADs. *California Probate Code* Sections 4600–4678 state that withholding or withdrawing healthcare is permitted “pursuant to an advance health care directive, by a surrogate, or as otherwise provided, so as to permit the natural process of dying.” Mercy killing or euthanasia are prohibited. *California Health and Safety Code* Sections 442–442.7 state that healthcare providers should offer comprehensive information and counselling regarding EOL care options. Finally, *California Probate Code* Section 4700–4701 gives provisions for an Advanced Health Care Directive.

In Germany, questions of EOL attracted major public attention with the case of physician Julius Hackethal promoting and practicing euthanasia in the mid-1980s. The issue was frequently discussed with reference to the crimes of physicians during the Nazi era. Religious groups also played an important part in the debate, especially the Roman Catholic Church and the Protestant Churches [27]. Both hold that euthanasia should be legally banned but support pain relief and accept withholding or withdrawing medical treatment in accordance with a patient’s wishes. In 2005, the German National Ethics Council issued recommendations for the legal regulation of EOL decisions, thus preparing the ground for the legislation on ADs in Germany. The report advocated the provision of basic care for all dying patients and emphasised the right to refuse treatment [18, 28]. The law on ADs (2009) [29] allows a variety of ADs as expressions of individual autonomy [30]. Any treatment decision has to respect an existing AD, irrespective of the stage of illness. Institutions like the Federal Ministry of Health, the medical associations, civil rights organizations, patient advocacy groups, and the Churches provide different forms of ADs which are all legally recognized. Physician-assisted suicide is not criminalized by German penal law, but there is an ongoing controversy among medical associations about its evaluation in light of professional ethics and law [31, 32]. Recent legislation has banned organized and commercial forms of assisted suicide but paved the way for single-case decisions.

In Israel, there is also a separate law on ADs, the *Dying Patient Act* (2006). It was the result of deliberations at the Public Committee on the Care of the Dying Patient, where different religious, ethical and professional perspectives were presented. According to the Orthodox Jewish (Halakhic) tradition, the sanctity of life has more ethical weight than individual autonomy. Active euthanasia is strictly forbidden. Withdrawing treatment is categorised as an active, life-shortening intervention and therefore deemed unacceptable. Withholding treatment, however, is considered as passive and thus permissible under certain conditions [28, 33, 34]. The *Dying Patient Act* regulates EOL care within the Jewish Halakhic restrictions on autonomy. ADs only apply within rather restrictive bureaucratic constraints [33–36]. In general, the Israeli law has two characteristics: First, the application of ADs is limited to terminal patients in the final 6 months of life; second, only withholding treatment is permissible. The interruption of an ongoing treatment, e.g., mechanical ventilation or artificial feeding, is not accepted, even if it is the patient’s will. As a compromise, a timer was proposed – a technical device turning a continuous treatment into a discontinuous one. Israeli ADs are completed on a complicated bureaucratic form issued by the Ministry of Health. Since the commencement of the act, the Ministry has received more than 7.200 ADs. However, many of them do not meet the complex formal requirements and are returned to the senders for correction (Eti Biton, registrar, Center for Advance Directives, Ministry of Health, personal communication January 1, 2017). A new potential development in Israel concerns the legalization of physician-assisted suicide. In June 2014, the Knesset’s Ministerial Committee for Legislative Affairs passed a bill legalizing physician-assisted suicide which was rejected in the Knesset in 2016. The law would have allowed doctors to administer a lethal injection to terminally ill patients in the last 6 months of their lives.

A common point of reference in all three countries’ debates is the Holocaust and the involvement of doctors in the Nazis’ programme of involuntary “euthanasia” [37]. In the US-American debate, drawing analogies to Nazism plays an important role in politically charged EOL controversies [38]. In the German discourse, the historical experience of atrocities committed by medical professionals is used as evidence supporting slippery-slope arguments against physician-assisted suicide or euthanasia. Remarkably, the views of Israeli Holocaust survivors regarding euthanasia (which only played a minor role in the legislation) are opposed to those of German professionals [39]. They argue that there are significant differences between the Nazis’ involuntary “euthanasia” programmes and physician-assisted suicide in accordance with a patient’s will. Cultural factors such as religion and the experience of the Holocaust also influence the attitudes of healthcare professionals [40] and the role of patient-support groups [41].

## Methods

In order to explore lay perceptions on EOL decision making, we conducted twelve focus groups (FGs) with religious (Jewish/Christian) and non-religious persons (personally affected and non-affected) in the United States (California), Germany, and Israel between 2010 and 2012. FGs are moderated group discussions with 6–10 participants. They are an established tool in qualitative research to explore common arguments and public topoi on a general level [42, 43]. The participants in our FGs were recruited by posters, flyers, and information sheets outlining the central aims and methods of the study and distributed in different public places. All interested persons responded voluntarily by submitting a flyer with their basic socio-demographic data and contact information. IRB approval had been obtained in advance from the Jewish Home San Francisco as well as San Francisco State University for the US groups, the University Medical Centre Göttingen for the German groups, and Ben-Gurion University for the Israeli groups.

Participants in the FGs were selected to reflect different contexts with regard to being affected and socio-economic background. “Being affected” was defined as having personal experience with EOL decision making, either due to own decisions or caring for a terminally ill friend or relative (compared to “non-affected” persons who had no such personal experience) [44]. In the US, two groups consisted of residents of the Jewish Home San Francisco and two of volunteers at the same facility. Participants in all US-groups identified as Jewish, but only roughly half of them as religious. The volunteers had primarily professional experiences with terminal care. In Germany and Israel, one non-affected group was composed of participants identifying as religious and the other of participants identifying as secular. The participants in the religious groups identified as Christian in Germany and as Jewish in Israel. Ultra-orthodox Jews were not represented in the Israeli sample. The participants in our FGs were by no means regarded as representative of the overall populations of the US, Germany, or Israel. Rather, we used the sample to explore how being religious or not interacts with other factors such as legal frameworks and historical backgrounds. For example, we were interested whether participants identifying as religious would refer to national policies or arguments of religious authorities in the respective public debates.

All FGs lasted between one and two hours. A semi-structured questionnaire with similar scenarios and questions was used. The discussions were audio recorded and transcribed. The German and Israeli FGs were translated into English to facilitate analysis. All material was pseudonymised using a number/letter code and later anonymized for publication (speakers are identified as M = male; F = female, as rel = religious, sec = secular, and as living in US = USA; GE = Germany, IL = Israel). Transcripts were coded using *Atlas.ti* software and analysed thematically in order to identify discursive themes and main lines of argument recurring within and across the groups [45, 46]. The focus was on moral perspectives on ADs, physician-assisted suicide, euthanasia, withholding and withdrawing life-sustaining treatment, and political regulation of EOL decision making [26]. To juxtapose lay moralities and the expert discourses, topics emerging through inductive coding were compared with the general categories of the bioethical discourse collected from analyses of public policies and expert committee statements [18, 28]. This helped to examine whether and how common ethical principles were embedded in public opinions and how they differed with regard to cultural factors in the context of EOL decision making.

## Results

The FGs had between 5 and 9 participants, with 82 participants (29 male and 53 female) in total, 23 in the US, 29 in Germany, and 30 in Israel. Participants were between 20 and over 90 years old. Their educational backgrounds differed, but the self-recruitment led to a slightly higher proportion of persons with an academic education.

A central topic of concern throughout all group discussions in all three countries was the meaning, scope, and limits of individual autonomy in EOL decision making. At the same time, however, autonomy was interpreted in different ways and different arguments were brought forward in favour of or against autonomous decision making in the different FGs, depending on varying situational aspects and socio-cultural contexts. The subsequent sections explore these similarities and differences with regard to ADs, termination of life-sustaining treatment and euthanasia, as well as the role of the state in EOL.

### A) Advance directives and planning EOL decisions

Participants in the German and the US FGs showed a rather strong tendency to emphasize the relevance of individual autonomy in EOL decision making, especially regarding the validity and binding character of ADs. In the corresponding discussions, different intellectual traditions of conceptualizing autonomy seemed to come into play. Thus, in line with a Kantian understanding of autonomy, a person’s will as put down in an AD was often considered as strictly “*sacrosanct*” (M-rel US), her right to self-determination as a matter of fundamental human dignity. At the same time, however, autonomous decisions did not necessarily have to be rational, but rather free from external interference in the sense of Anglo-American liberalism. In this vein, an individual’s autonomous decision was, for example, supposed to trump all familial and professional reservations. Thus, combining both aspects, many speakers were convinced

*that the will of the patient should be determining, regardless what the doctors say; regardless what the relatives say. The patient has decided that when he was still healthy and of sound mind. One would go over his head and act against his will if one would do something else than what he has written down. And I believe that this is part of the dignity of the human person, and of the […] self-determination of humans, that he can decide what he wants* (F-sec GE).

Furthermore, especially in the German FGs*,* many speakers expressed the conviction that there is not only a right, but actually a moral responsibility towards one’s next of kin or attending physicians to hold an AD. Individuals were expected to take care of their own EOL matters, that is, to make up their mind, come to a clear decision, and finally complete “*a precise document*” in order not to “*burden another person with this responsibility*” (F-sec GE) of making serious proxy decisions regarding other people’s life and death.

The Israeli groups, by comparison, were much more reluctant and ambivalent in this regard. Participants frequently suggested that the application of ADs has to be coordinated with other formal requirements including a restriction of the options that can be chosen. However, there were additional differences underlying this “Israeli” stance. Secular speakers mainly discussed the tension between individual self-determination and moral responsibilities towards others, e.g., relatives or attending physicians. The latter should not be burdened with executing problematic directives, especially if they have lethal consequences. The religious speakers, on the other hand, also pointed out further preconditions and limitations of individual self-determination, such as divine will, creation, and the natural order of things:

*Many religious people believe that the time of death is written – that God knows when death will occur, and humans shouldn’t interfere. If that is true, how can anyone know when medical life support is appropriate, or when it merely prolongs dying? It is futile to man, but on the other hand – we are not the ones to do the work of nature, it would be taking a role that is conflicting with the creation and nature. (M-rel IL)*

In contrast to the secular speakers, many religious participants saw “*a great difference*” *(F-rel GE)* between withholding and withdrawing medical treatment when it comes to ADs. The underlying intuition seemed to be that a purely consequentialist, outcome-oriented moral perspective ignores that it makes *“a major difference if I omit something, or if I act directly*” *(F-rel GE).* Especially the Israeli religious participants were practically unanimous in this respect. In consequence, they frequently contested the validity of ADs if these demanded a withdrawal of life support. Such demand was considered illegitimate and repeatedly equated with *“promoting suicide*” or even killing:

*I agree, the directives in this case are not binding for the doctors. Generally speaking […], nobody can determine the fate of another person. In this case particularly – disconnecting from life support machines is the same as killing. It is legally forbidden and it is prohibited by Judaism. (M-rel IL)*

Our findings thus indicate a certain tension between a secular emphasis on individual autonomy and more comprehensive religious perspectives. Thus, while many secular participants defended autonomy in the sense of unhampered self-determination, religious participants often complemented and weighed individual self-determination with other aspects such as responsibility for others or respect for divine creation or natural law. Furthermore, the distinction between withholding and withdrawing treatment played a decisive role in this context. Interestingly, however, it did not seem to be exclusively linked to Judaism as could be expected against the backdrop of the corresponding Halakhic teachings. Thus, while the participants at the Jewish Home San Francisco unanimously rejected the idea that there is a morally significant difference between withholding and withdrawing, the other religious speakers, including the Christian ones in Germany, expressed the sense that this difference should be taken into account.

### B) Termination of life-sustaining treatment and euthanasia

We also found a complex array of moral stances towards termination of life-sustaining treatment and euthanasia amongst our respondents. In line with a liberal understanding of autonomy, many participants in the German and US-groups applied the right to self-determination to include withholding or withdrawing life-sustaining treatment. In the groups of aged residents at the Jewish Home San Francisco, this attitude was also backed by a sense of urgency due to being personally affected:

*I believe in … euthanasia. … I have had so much experience in my own family with death directly, uh, to the point of uh, holding my sister when she died, and feeling her life spirit leave her body, that my attitude has changed a great deal on that score. … I think it’s very important even that the families change their attitudes and let people go (F-rel US).*

By contrast, especially in the German groups, moral responsibilities towards other actors involved, such as relatives or medical professionals, were frequently weighed in and played out against individual self-determination. The underlying concern was that it is not legitimate to burden other persons with the responsibility of executing one’s own dying wish:

*But one must also consider who can be burdened with that. I think, well even if one could talk about it with someone, a relative: “I want this and this not so,” but then one still has to stand up for this decision before the doctor: “Please turn the machine off, he shall die.” This is indeed a heavy burden. To burden one person with all of that … (F-sec GE).*

The Israeli FGs generally expressed strong reservations against euthanasia. Thus, especially the religious speakers were strictly opposed to euthanasia and – although to a lesser degree – also to termination of life-sustaining treatment. Theological doctrines and interpretations figured prominently as a basis for the arguments expressed. Thus, for many Jewish Israeli respondents, life is given and taken by God, so that human decisions and actions are not allowed to interfere:

*According to Jewish religion it is forbidden to hasten death, that would be equivalent to murder, and allowing doctors to decide about hastening death can lead to incorrect decisions and recklessness bordering on murder. M-rel IL: Yes, I also agree with this prohibition … We have to let nature take its course. (M-rel IL)*

It is important to note, though, that the opposition to euthanasia was not necessarily attributed to Jewish religion or theology in a narrow sense, but often also to a broader notion of Jewish cultural identity. Thus, the idea of ending life was repeatedly described as being alien to one’s own cultural tradition and values, with one Israeli Jewish speaker finding it “*actually quite curious”* that “*this concept never invaded Jewish thought, even though Jewish people suffered throughout history” (M-rel IL).* This clear idea of a historical and cultural Jewish identity played an important role in the Israeli FGs. Thus, while the FG participants at the Jewish Home San Francisco articulated a rather lenient understanding of being Jewish – “*you talk to … ten Jews and they probably have ten different ideas about that*” *(F-rel US)* – the Israeli groups tended to formulate a much more compact, homogeneous conception:

*As Jews, modern religious or otherwise, we should value life and respect our bodies. Furthermore, we have the responsibility to care for ourselves and seek medical treatment needed for our recovery-we owe that at least to ourselves, to our loved ones, and to God. (M-rel IL)*

Interestingly, in the German groups, references to the country’s Nazi past seemed to play a similar role. They created a presumed cultural identity which precluded overly liberal stances towards euthanasia. In this sense, the following speaker alluded to the historical Nazi “euthanasia”-programmes in a slightly embarrassed manner:

*I believe if one is thinking now that they possibly continue to live disabled, then this is actually…That will pretty soon go into the direction of [laughs] Auschwitz. Well, this is what the Nazis wanted too. That is only healthy people and so on. Life is not this way. (F-sec GE)*

Our participants’ responses to the moral problem of euthanasia confirmed the impression that attitudes towards EOL decision making do not so much depend on a specific “culture” and “religion” as such, but on many different and interlaced factors. Thus, a general idea of individual autonomy played a major role for participants in both the US and German groups, religious or otherwise. This stance could be supported by the perspective of being personally affected through advanced age or ailing health. At the same time, however, the idea of responsibility for others, as well as lessons learnt from national history such as the Nazi euthanasia programme suggesting slippery slope effects, could act as a counterbalance against overly individualistic and liberalistic interpretations of autonomy as unrestricted personal self-determination. Once again, participants in the Israeli groups – religious as well as secular – drew a somewhat different picture with regard to euthanasia. But even there, opposition to euthanasia was not based on theological arguments alone (not even in the religious group), but rather on the cultural interpretation of Jewish identity as centring on a high valuation of life.

### C) The role of the state regarding EOL

When it comes to the question how the state and political or legal regulations should deal with EOL decision making, rather clear-cut national differences emerged in the FGs. Thus, in the German groups, the state was mainly viewed as a neutral authority which should set general legal framework conditions, but stay out of concrete decision making processes regarding ADs or EOL situations. The state “*should draw up a recommendation”* but “*must not exert pressure*” (F-sec GE). Respecting highly individual decisions, e.g., regarding the format of ADs, was also expected:

*Death is not a bureaucratic act, and neither is the way to it. And correspondingly, I would plead to keep the state as far as possible out of it. It can issue recommendations. It can lend a helping hand, especially with respect to such lists. …But otherwise it should stay out of it. Of course we want to make life easier for our doctors, but the patient comes first. (M-sec GER) It is ok for the state not to have a say. This is actually a condition. (M-sec GE)*

In addition, the participants in the German FGs discussed the political regulation of EOL-issues in a European context. Thus, they compared the German setting to the situation in the Netherlands or Switzerland which have more permissive legal regulations regarding assisted suicide or euthanasia. Participants also discussed issues of euthanasia tourism and the different legal and juridical situation between the national and the European levels, addressing the complicated questions arising from transnational standardisation:

*There is a woman, who … was in a condition that she could not react or decide anymore. There existed an advance directive, but the husband did not want to switch off. He wanted that she will be helped and is given a certain medication. … And this was not possible in Germany. … Now he travelled with her to Switzerland where she, so to say, was put to sleep. And now he sued at the Federal Court of Justice, which has dismissed the suit, but the European Court of Justice has accepted it. Now it will be decided, if the doctors acted here wrongly in refusing this euthanasia. (F-sec GE)*

The Israeli FGs reserved a much more central role for the national state, albeit with completely different motives: The secular speakers put great trust in the state and its bodies (especially courts) as neutral, rational authorities which are above arbitrary individual preferences as well as partisan quarrels. The state “*functions to maintain order and protect its citizens, and not leave it to the whims of everyone*” *(F-sec IL).* It has the power to defend individual life and rights against majority pressure and religious influence and to enforce generally binding rules. In consequence, its impartial courts should decide in cases of conflict and doubt:

*You need to consider all views and take into account all the contexts of a situation. Probably every one of those who are involved will have arguments that are very convincing. In the end who I think need to decide – it’s the court because the court looks at things objectively – or at least that’s what it should do. (F-sec IL)*

The Israeli religious speakers, on the other hand, articulated the almost diametric view that the Israeli state indeed embodies a substantial religious stance. Many participants expressed a rather strong belief that state regulations regarding EOL decision making are legitimate since (and inasmuch as) they are actually in accordance with Jewish law (Halakha). Some participants were convinced that the national “*law was made in agreement with the rabbis; otherwise it would not have become a law*” *(M-rel IL).* This was perceived as a relieving reassurance that legally permissible decisions will also be morally irreproachable.

By contrast, the attitudes in the US FGs at the Jewish Home San Francisco showed much more reservation towards the state. Speakers repeatedly expressed the experience of being members of a religious or cultural minority in a political culture dominated by Christianity. The US was perceived as “*primarily a Christian country*” *(F-rel US)* which has also an impact on political regulations of EOL decision making “*because here in the United States, we want to protect life, so much, that … you’re going to have some difficulty…, especially when it comes to … suggesting changes*” *(F-sec US)*. Since the mainstream Christian culture was seen as influencing political decisions and legal regulations that affect one’s own personal situation at the end of life, there was concern about ensuring individual autonomy and personal rights in EOL decision making among the Jewish Home residents:

*That’s just as important as how you end your life, having the right to end it. Unfortunately, we don’t have that right. Not legally anyway. And sometimes I worry about that. Cause I live with a lot of pain that I manage, but what if I couldn’t manage it? Would I be able to say I don’t wanna live anymore please say goodbye, I wanna call my family? No! I don’t have that right. That worries me, I wish I could go to Congress and change the laws. Or find a new Dr. Kevorkian to come into the world. [CHUCKLES] (F-sec US)*

Maybe due to the experience of being marginalized in a predominantly Christian culture, there was a strong awareness of the cultural aspects and differences of attitudes towards EOL in the US groups. In Israel, the Jewish homeland, the strong nexus between religion and nationality could explain the support and trust expressed by many of the Israeli respondents toward the role of the state. By contrast, the US experience could have led to a sense of alienation from mainstream culture and the political governance of EOL. Indeed, this sense was sometimes articulated by way of grim sarcasm in view of the perceived perplexities and inconsistencies of the majority position:

*I mean um, Christians live to go to Heaven, and why, why is there such a fear of death? When Terry Schiavo died that was a big political case here, that was what, that’s what got me to do my first advance directive! And, as soon as she died, her brother went on the air, who was totally against pulling the tubes and said, “Well, she’s in a better place now.” […]: What’s wrong with this picture?! Why wouldn’t you let her go there, if she’s in a better place? (F-rel US)*

## Discussion: taking culture and religion seriously

Our findings draw attention to the variety and complexity of cultural and religious aspects of EOL decision making. In contrast to stereotypical prejudices, e.g., about rational secularists and unthinking “religionists,” moral reasoning among those identifying as religious often proved to be rather varied and nuanced. Narrow interpretations of individual autonomy in the sense of personal self-determination were complemented with and sometimes relativised by other aspects, such as responsibility for others, natural law, or divine will. Furthermore, purely consequentialist views levelling the moral relevance between withholding and withdrawing treatment were put in perspective.

At the same time, however, the group discussions indicate that there are usually no clear-cut positions anchored in “nationality,” “culture,” or even “religion”; rather, attitudes are personally decided on as part of a negotiated context representing the specific political, social, and existential situation of the individual. Although there is local consensus that goes beyond radical individualism, cultural specificities and differences in this field generally do not seem to be neatly matched with national cultures or religious denominations. Instead, the fundamental relevance of the specific *situatedness* of religious beliefs and cultural communities becomes visible: Their status and role in individual situations, for example, as consensual or conflicting on the level of personal perspectives, family relationships, and broader social contexts, e.g., as a majority or minority culture within a political system (Table 1).

**Table 1:** Synopsis of public attitudes in US, GE, and IL

Speakers		US		GE		IL	
Topics		*Sec*	*Rel*	*Sec*	*Rel*	*Sec*	*Rel*
*ADs*		Respect of ADs as expression of personal autonomy	Respect of ADs as expression of personal autonomy	- Respect of ADs as expression of personal autonomy - Responsibility towards relatives and doctors to have an AD	Respect of ADs as expression of personal autonomy	Reluctance towards ADs due to moral responsibilities towards others	Reluctance towards ADs due to transcendental aspects (divine will, creation, natural order)
*Withholding/withdrawing*		No significance	No significance	No significance	Significance	Some significance	Strong significance
*Euthanasia*		Mixed/Acceptance due to individual autonomy and being affected	Mixed/Acceptance due to individual autonomy and being affected	Mixed/Reluctance on basis of responsibility towards others and Nazi past	Mixed/Reluctance on basis of responsibility towards others and Nazi past	Reservations on basis of Jewish identity	Opposition on basis of Jewish identity and divine will
*Role of state*		Negative role as representing Christian religion limiting personal autonomy	Negative role as representing Christian religion limiting personal autonomy	Positive role as neutral authority respecting individual autonomy	Positive role as neutral authority respecting individual autonomy	Positive role as neutral authority respecting individual autonomy	Positive role as representing Jewish law

Of course, the specific qualitative design of our study and the composition of the samples have limitations that need to be taken into account when interpreting the findings. Thus, we only conducted group discussions with secular, Jewish, and Christian persons in Germany, Israel, and the US (only in California). Moreover, there was a slight bias towards higher education which might have influenced the way EOL-issues were perceived and discussed. Finally, our sample is not symmetrical as it does not include Jewish persons in Germany or Christian persons in the USA or Israel. Further, more systematic empirical research will be needed in order to draw a richer and more differentiated picture of the respective influences of and interactions between the religious commitments and the national situatedness of participants. Moreover, similar studies including additional countries and denominations could help to explore whether laypeople from other national or religious backgrounds and in different sociocultural settings would express divergent perspectives on EOL, e.g., Muslims in the US, Israel, and Germany (for an Islamic theological perspective on EOL, see [23]).

## Conclusions: towards a comparative empirical bioethics

On a *practical ethical level*, our findings suggest that there are good reasons for being suspicious of academic positions, professional stances, and practical guidelines that seek to derive persons’ moral attitudes towards EOL mainly from their cultural or religious backgrounds. Cultural and religious perspectives seem to be far too complicated to predict individual preferences regarding limitation of care or euthanasia. As our findings show, this may not just be a pragmatic problem of dealing with *complexity* in the sense that we need more statistical information about additional factors potentially modifying cultural or religious convictions. Indeed, no statistical model, however complex, seems to do justice to the fundamental *reflexivity* of modern cultural and religious attitudes: the fact that people not just belong to certain cultural or religious communities, but frequently position themselves in relation to their respective commitments by interpreting, reviewing, modifying, or even abandoning them. In this sense, our speakers often explicitly articulate their own stances as “Christian” or “Jewish” while at the same time qualifying them with reference to particular authoritative traditions or cultural circumstances, e.g., as a Jewish minority in a Christian majority culture. Thus, rather than going through great pains to develop elaborate decision making algorithms, our findings suggest that the conditions and standards of actual decision making need to be improved. One important way of doing so would be by strengthening the reflexive and communicative practice of completing ADs [47], as newer efforts of so called Advance Healthcare Planning conceptualize this rather as process than as a fixed document [48].

On a *theoretical level*, these findings can provide an important clue for a contemporary bioethics that is willing to engage seriously with cultural and religious diversity. It suggests that the discipline has to be informed by adequate theoretical conceptions *and* empirical insights. In this sense, culturally informed bioethics should not rely on a do-it-yourself approach to culture, but systematically take into account the approaches and results of contemporary cultural theory. This can help to question essentialist and monolithic conceptions in discussions about EOL decision making. Furthermore, it facilitates the differentiation between various factors that play into “culture,” as well as the analysis of their dynamic interaction and development, e.g., effects of migration, acculturation, collective history, family story, and individual biography [49, 50]. The same holds true in view of the religious dimension: In order to develop an adequate understanding of religion and avoid stereotyping, bioethical theory needs to include the perspectives and insights of religious studies. They can help to differentiate between diverging interpretations of religious sources like the Bible or the Halakha, and to distinguish authoritative theological positions from lived religious experience and practice influencing personal attitudes, e.g., in the context of cultural Judaism [51]. Hence, cooperation between bioethics and religious scholars can be fruitful when it comes to working out a more fine grained understanding of religious factors in bioethical decision making processes [52].

The theoretical implications of this study thus encourage bioethics to develop in a direction that can be called “comparative empirical bioethics” [53]. In contrast to the classical division between deductive and inductive approaches in bioethics, this approach uses both, narrative, empirically induced findings about moral experience as well as theoretically informed categories to understand and critically reflect moral attitudes. A major attempt is to understand, analyse, and reflect on “implicit” assumptions made in everyday life morality as well as in expert statements. This methodology helps to flesh out some parts of the “whole” picture, while others still remain vague and need to be examined. Here, we focused mainly on religion, but other studies might evidently focus on the empirical implications of ethnicity, gender, patient identity, or their intersection. This is exactly the kind of inductive uncertainty which makes empirical bioethics so difficult to embrace for a deductive approach, but also a necessary challenge to test theoretical pre-assumptions.

## Abbreviations

AD: Advance directive
EOL: End-of-life
FG: Focus group
GE: Germany
IL: Israel
Rel: Religious
Sec: Secular
US: USA
